# Rhein Suppresses Neuroinflammation via Multiple Signaling Pathways in LPS-Stimulated BV2 Microglia Cells

**DOI:** 10.1155/2020/7210627

**Published:** 2020-06-29

**Authors:** Piao Zheng, Xuefei Tian, Wei Zhang, Zhaoyu Yang, Jing Zhou, Jun Zheng, Hanjin Cui, Tao Tang, Jiekun Luo, Yang Wang

**Affiliations:** ^1^Institute of Integrative Medicine, Department of Integrated Traditional Chinese and Western Medicine, Xiangya Hospital, Central South University, Changsha, Hunan 410008, China; ^2^College of Integrated Traditional Chinese and Western Medicine, Hunan University of Chinese Medicine, Changsha, Hunan 410208, China; ^3^Shanxi Province Hospital of Traditional Chinese Medicine, Shanxi Provincial Institute of Traditional Chinese Medicine, Taiyuan, Shanxi 030012, China; ^4^College of Chemistry and Chemical Engineering, Central South University, Changsha, Hunan 410083, China

## Abstract

As a bioactive absorbed compound of rhubarb, Rhein is applied for the treatment of brain injury. However, the underlying pharmacological mechanisms remain unclear. In this study, we aimed to explore antineuroinflammatory functions and underlying mechanisms of Rhein in vitro. BV2 microglia cells were chosen and irritated by LPS. The influence of Rhein on cell viability was determined using MTT assay. We finely gauged the proinflammatory cytokines of TNF-*α* and IL-1*β* through tests of immunofluorescence staining, ELISA, RT-qPCR, and western blot. Additionally, mediators including IL-6, IL-12, iNOS, and IL-10 were surveyed by ELISA. Furthermore, protein levels of the underlying signaling pathways (PI3K/Akt, p38, ERK1/2, and TLR4/NF-*κ*B) were tested adopting western blot. We found that Rhein reduced the secretion of pivotal indicators including TNF-*α* and IL-1*β*, effectively restraining their mRNA and protein expression in LPS-activated BV2 microglial cells. Besides, Rhein treatment demoted the production of IL-6, IL-12, and iNOS and promoted the excretion of IL-10. Subsequent mechanistic experiments revealed that Rhein obviously downregulated the phosphorylation levels of PI3K, Akt, p38, and ERK1/2 and simultaneously upregulated the PTEN expression. In addition, Rhein antagonized the increase of TLR4, p-I*κ*B*α*, and NF-*κ*B. In summary, Rhein suppresses neuroinflammation via multiple signaling pathways (PI3K/Akt, p38, ERK1/2, and TLR4/NF-*κ*B) in LPS-stimulated BV2 microglia cells. This study highlights a natural agent for prevention and treatment of neuroinflammation.

## 1. Introduction

Central nervous system (CNS) controls physical and mental functions. Its collapse causes neurological and neurodegenerative diseases such as traumatic brain injury (TBI), stroke, Parkinson's diseases, multiple sclerosis (MS), and amyotrophic lateral sclerosis (ALS). Most of these diseases are severe and incorrigible due to extremely limited regenerative potential of CNS [1]. CNS injury involves a series of complex pathological processes. Neurons, glial cells, and endothelial cells participate in these processes and produce immune medium. Of these cells, microglia mainly contribute to the neuroinflammation which plays an important role in brain damage [2]. CNS releases a large number of inflammatory immune factors after injury, which mediates a series of waterfall-like inflammatory responses. The responses can accelerate pathological progression and worsen subsequent prognosis [3]. Therefore, to improve or reverse the pathological process through inhibiting neuroinflammatory responses becomes a coincident strategy to treat neurological diseases.

Although the studies of antineuroinflammatory drugs obtain promising results [4], the clinical application is disappointing [5]. Pharmacologists and doctors believe that novel chemicals or therapeutic agents to treat neuroinflammation are urgent. Fortunately, 34% of these molecules are from herbal medicines or their direct derivatives [6]. Scientists hope to search active ingredients originating from herbal medicines as promising agents for neuroinflammatory treatment.

As the main anthraquinone compound of traditional Chinese medicine rhubarb, Rhein is utilized in a wide-range, owing to multitudinous pharmacological activities embracing laxative, anticancer, antidiabetic, and antibacterial effects [7]. Our previous studies found that Rhein is the only anthraquinone entering the blood-brain barrier (BBB) and gives neuroprotection similar to rhubarb [8, 9]. Moreover, the potential anti-inflammatory properties of Rhein in multiple disease models are identified [10, 11]. However, it is unclear whether Rhein exerts anti-inflammatory effects in CNS diseases. In addition, its underlying molecular mechanisms remain vague.

The most prominent secondary lesion in the CNS injury is neuroinflammation [12, 13]. As an immune sentinel for almost all inflammatory reactions in the CNS, microglia recognize triggers of neuroinflammation through a batch of receptors on it to achieve activation and proliferation. Activated microglia boost the release of cytokines and chemokines, containing proinflammatory cytokines, reactive oxygen species, and metalloproteinases [14]. The proinflammatory cytokine interleukin-1 (IL-1) entices the emittance of reactive oxygen species and metalloproteinases from neurotoxic mediators. Tumor necrosis factor promotes proteolytic enzyme release, BBB breakdown, and induction of cell death. This factor tends to initiate secondary damage to the CNS [15–17]. Vasodilation and plasma extravasation in secondary injury provoke the release of neuropeptide, drive the interactions of leukocyte migration and adhesion molecules, and further actuate the production of histamine and cytokine IL-6 [18]. Afterwards, the interplay between these molecules contributes to neurological dysfunction [19].

Thus, we aimed to ascertain whether Rhein resists neuroinflammation and further to investigate its potential multiple molecular mechanisms. The present study will help to clarify the potential therapeutic significance of Rhein for neuroinflammatory therapy.

## 2. Materials and Methods

### 2.1. Chemicals

Rhein, the purity by authorization >98%, was gained on the National Institute for the Control of Pharmaceutical and Biological Products (Beijing, China). Dulbecco's Modified Eagle Medium (DMEM) and fetal bovine serum were attained from Thermo Fisher Scientific (Waltham, MA, USA). Phosphate Buffered Saline (PBS) was gained on HyClone (Logan, Utah, USA). LPS, dissolved in dimethyl sulfoxide (DMSO) and 3-(4,5-dimethylthiazol-2-yl)-2, 5-diphenyl tetrazolium bromide (MTT) were acquired from Sigma-Aldrich Biotechnology (St. Louis, MO, USA). Tumor necrosis factor-alpha (TNF-*α*) anti-mouse monoclonal antibody, interleukin-1 beta (IL-1*β*) anti-rabbit polyclonal antibody, Alexa Fluor 594-conjugated Goat Anti-Mouse IgG (H + *L*), and Alexa Fluor 488-conjugated AffiniPure Goat Anti-Rabbit IgG (H + *L*) were brought from Proteintech (Danvers, MA, USA) and 4′6-diamidino-2-pheylindole (DAPI) was purchased from Sigma-Aldrich Biotechnology (St. Louis, MO, USA). Enzyme-linked immunosorbent assay (ELISA) kits of TNF-*α*, IL-1*β*, interleukin-6 (IL-6), interleukin-10 (IL-10), and interleukin-12 (IL-12) inducible nitric oxide synthase (iNOS) were supplied by Cusabio Technology (Wuhan, Hubei, China). Antibodies against IL-1*β*, TNF-*α*, *β*-actin, p38, Toll-like receptor 4 (TLR4), and glyceraldehyde phosphate dehydrogenase (GAPDH) were purchased from Proteintech (Danvers, MA, USA). Antibodies against phosphoinositide 3-kinase (PI3K), phosphatase and tensin homolog (PTEN), extracellular signal-regulated kinase 1/2 (ERK1/2), and phosphorylated-p38 (p-p38) were received from Abcam (Cambridge, MA, USA). p-PI3K, protein kinase B (Akt), p-Akt, p-ERK1/2, phosphorylated I kappa B-alpha (p-I*κ*B*α*), NF-*κ*B p65, and Lamin B were from Cell Signaling Technology (Beverley, MA, USA). Horseradish peroxidase (HRP)-conjugated secondary antibodies were from Proteintech (Danvers, MA, USA). All the other reagents were of analytical grade.

### 2.2. Microglial Cell Culture and Treatment

The mouse BV2 microglial cell line was purchased from Institute of Basic Medicine, Chinese Academy of Medical Sciences (Beijing, China). BV2 cells were supplemented with 100 U/mL penicillin, 10% heat-inactivated fetal bovine serum, and 100 *μ*g/mL streptomycin solution, using DMEM as supporter. They were cultured in a humidified incubator with 5.0% CO2 at 37°C. To measure the anti-inflammatory effects of Rhein, BV2 cells were treated with Rhein 30 min before LPS was added.

### 2.3. Cell Viability

We used MTT assay to evaluate the viability of BV2 cells after treatment of Rhein. The cells were cultured in 96-well plates at a density of 1 × 10^4^ cells per well. Different concentrations of Rhein (0–160 *μ*M) were added and the remaining wells were filled with PBS. After treatment for 48 h in the incubator, cells were incubated in 10 *μ*L MTT solution 10 *μ*L for 4 h and then 150 *μ*L DMSO was added for 10 min to dissolve the crystals. Viabilities were determined at 490 nm.

### 2.4. Immunofluorescence Staining

After various treatment, BV2 cells were seeded on glass coverslips and then fixed for 20 min with 4% paraformaldehyde followed by permeation for another 30 min at 37°C with 0.3% Triton X-100. The cells were blocked for 1 h with 5% bovine serum albumin (BSA) to avoid nonspecific staining and incubated in TNF-*α* primary antibody (1 : 50) and IL-1*β* primary antibody (1 : 50) at 4°C overnight. Further, Alexa Fluor 594-conjugated Goat Anti-Mouse IgG (1 : 1000) and Alexa Fluor 488-conjugated AffiniPure Goat Anti-Rabbit IgG were incubated at room temperate for 90 min as second antibodies. The cells were finally incubated with DAPI to stain nucleus. Fluorescent staining and images catching were completed under positive fluorescence microscope (Motic, Fujian, China).

### 2.5. ELISA Test

The supernatants of BV2 cells were obtained after intervention. According to the manufacturer's protocols, ELISA kits were used to quantify the level of TNF-*α*, IL-1*β*, IL-6, IL-10, IL-12, and iNOS in the culture media.

### 2.6. Reverse Transcription Polymerase Chain Reaction (RT-qPCR) Assay

Total RNA was extracted from all BV2 cells using the TRIzol reagent (Invitrogen, Waltham, MA, USA), and the purity of RNA was measured by ultraviolet spectrophotometer (Molecular Devices, Sunnyvale, CA, USA). With the RNA reverse transcription kit (Cwbiotech, Beijing, China), total RNA was reverse transcribed to cDNA. Then SYBR green chemistry was used to quantified relative mRNA levels. QuantStudio 6 Flex Real-Time PCR System (Thermo Fisher Scientific, Waltham, MA, USA) was applied. Meanwhile, reaction conditions were 95°C for 10 min, followed by 40 cycles at 95°C for 15 s, at 60°C for 60 s. Primer sequences are as followed: TNF-*α* (product length 230 bp) forward 5′-TCCAGGCGGTGCCTATGTC-3′; reverse 5′-TCCTCCACTTGGTGGTTTGC-3′. IL-1*β* (product length 206 bp) forward 5′-CGTTCCCATTAGACAACTGCA-3′; reverse 5′-GGTATAGATTCTTTCCTTTGAGGC-3′. *β*-Actin (product length 116 bp) forward 5′- CATCCTGCGTCTGGACCTGG-3′; reverse 5′- TAATGTCACGCACGATTTCC-3′.

### 2.7. Western Blot (WB)

Whole protein lysates were prepared in radio-immunoprecipitation assay (RIPA) lysis buffer (Applygen Technologies Inc., Beijing, China). Nuclear and cytoplasmic proteins were extracted from Nuclear and Cytoplasmic Protein Extract Kit (Thermo Fisher Scientific, Waltham, MA, USA). Protein concentrations were measured choosing bicinchoninic acid (BCA) protein assay kit. The protein samples were loaded on 10% sodium dodecyl sulfate-polyacrylamide gels (SDS-PAGE) and separated through electrophoresis and then blotted on polyvinylidene fluoride (PVDF) membranes (Millipore, Billerica, MA, USA). After this, with 5% nonfat milk in Tris-buffered saline and Tween 20 (TBST) (Sigma-Aldrich Biotechnology, St. Louis, MO, USA), the membranes were blocked for 1 h at room temperature, followed by incubation at 4°C overnight with primary antibodies specific to TNF-*α* (1 : 1000), IL-1*β* (1 : 500), *β*-actin (1 : 1000), PI3K (1 : 1000), p-PI3K (1 : 1000), Akt (1 : 1000), p-Akt (1 : 2000), PTEN (1 : 1000), p38 (1 : 200), p-p38 (1 : 1000), ERK1/2 (1 : 10000), p-ERK1/2 (1 : 2000), TLR4 (1 : 500), p-I*κ*B*α* (1 : 1000), GAPDH (1 : 2000), NF-*κ*B p65 (1 : 1000), and Lamin B (1 : 5000). Following through washing three times with TBST, HRP-conjugated secondary antibodies (1 : 3000) were applied to incubate with membranes. The blots were developed by the use of Super Enhanced Chemiluminescence Detection Kit (Thermo Fisher Scientific, Waltham, MA, USA) with Bio-Rad ChemiDoc XRS^+^ System (Bio-Rad Laboratories, Inc., Hercules, CA, USA).

### 2.8. Statistical Analyses

Quantitative data are presented as the mean and standard error of the mean (mean ± SEM). Significant differences were determined by a one-way analysis of variance (ANOVA) followed by Turkey's post hoc test. A *p* value of less than 0.05 (*p* < 0.05) was considered significantly different. All statistical tests were carried out with GraphPad Prime 7 statistical analysis software (GraphPad Software Inc., La Jolla, CA, USA).

## 3. Results

### 3.1. Effects of Rhein on Cell Viability in BV2 Microglial Cells

**Figure 1:**
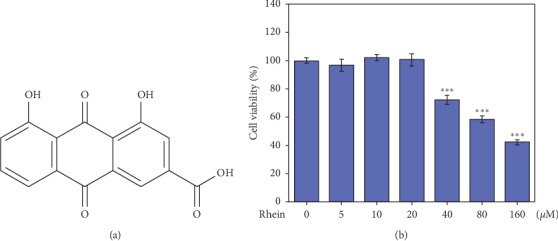
Effects of Rhein on the cell viability of BV2 cells. The cells were incubated with different doses of Rhein for 48 h and the cell viability was evaluated by MTT assay. Data represent the mean ± SEM of three independent experiments; each experiment was performed in triplicate. ^*∗∗∗*^*p* < 0.001 vs. control.

To estimate the effects of Rhein on cell viability in BV2 microglial cells, we incubated BV2 cells with varied concentration of Rhein for 48 h and then used MTT assay. As shown in Figure 1, 0–20 *μ*M of Rhein did not induced obvious effects on viability of BV2 microglial cells. With the dose increased, Rhein remarkably decreased the viability of BV2 microglial cells. 160 *μ*M of Rhein almost reduced the cell viability by 40% compared with control group. According to MTT results, we selected 3 *μ*M and 15 *μ*M as the low and high doses of Rhein, respectively, in the subsequent experiments.

### 3.2. Effects of Rhein on TNF-*α* and IL-1*β* in LPS-Stimulated BV2 Microglial Cells

The prophase neuroinflammation is caused by cytokines and neurotoxic factors secreted by microglia [20], in which TNF-*α* and IL-1*β* play crucial roles [21, 22]. To prove the inhibitory effects of Rhein on neuroinflammation, we mainly focused on the effects of LPS-stimulated BV2 microglial cells induced on proinflammatory factors including TNF-*α* and IL-1*β*.

**Figure 2:**
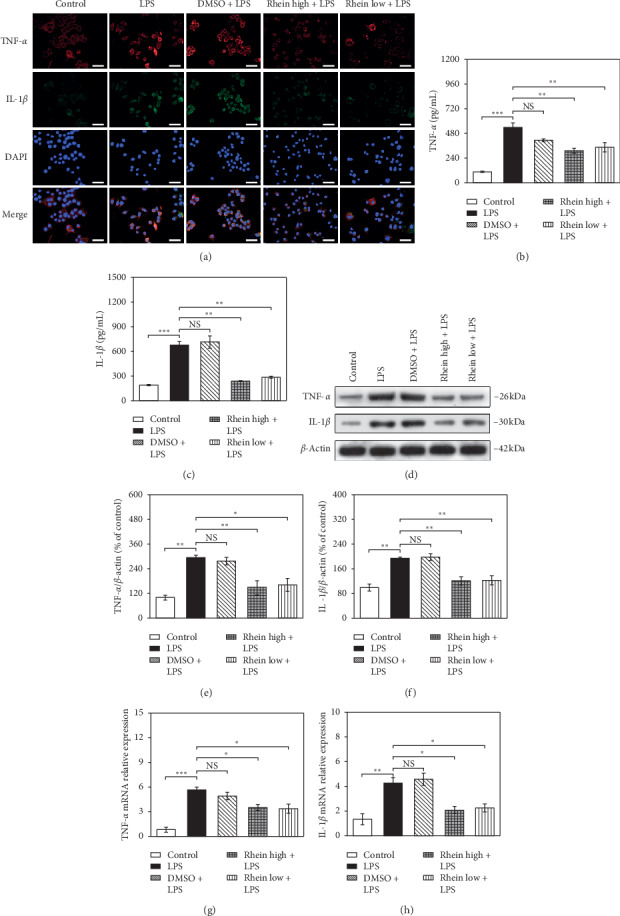
Effects of Rhein on TNF-*α* and IL-1*β* in LPS-stimulated BV2 cells. The cells were untreated (control) or pretreated with Rhein/DMSO for 30 min, followed by LPS stimulation (1 *μ*g/mL) for 48 h. (a) Immunofluorescence staining of cells was indicated by TNF-*α* antibody (red) and IL-1*β* antibody (green). DAPI (blue) was stained for visualization of nuclei. (b, c) The ELISA result histogram of TNF-*α* and IL-1*β*. (d) The WB bands (e, f) The WB result histogram of TNF-*α* and IL-1*β*. (g, h) Effects of Rhein on mRNA levels of TNF-α and IL-1β in LPS-stimulated BV2 cells. The mRNA expression was analyzed using RT-qPCR, with *β*-actin as reference gene. Data represent the mean ± SEM of three independent experiments. ^*∗*^*p* < 0.05, ^*∗∗*^*p* < 0.01, and ^*∗∗∗*^*p* < 0.001.

Immunofluorescence double-labelled images (Figure 2(a)) revealed that IL-1*β* (green) and TNF-*α* (red) fluorescence expression was obviously enhanced in the LPS-stimulated BV2 cells compared with the control group. Also, the fluorescence intensity of DMSO + LPS group was accorded with LPS group, indicating that the addition of DMSO had no effect on the immunofluorescence results. When compared with LPS group, TNF-*α* (red) and IL-1*β* (green) fluorescence signals were significantly decreased after Rhein treatment (high and low does). These phenomena were further confirmed by the ELISA results. As shown in Figures 2(b) and 2(c), the secretion standards of TNF-*α* and IL-1*β* in the Rhein groups were visible inferior to that in the LPS group (*p* < 0.01).

Next, the WB results demonstrated that Rhein (high and low does) efficaciously reduced the expression of TNF-*α* and IL-1*β* in contrast with LPS group (Figures 2(d)–2(f)). RT-qPCR was simultaneously adopted for investigation. The results of Figures 2(g) and 2(h) displayed that the TNF-*α* and IL-1*β* mRNA levels were downregulated by Rhein (high and low does) (*p* < 0.05).

### 3.3. Effects of Rhein on Cytokine Mediators and Neurotoxic Factors Including IL-6, IL-12, iNOS, and IL-10 in LPS-Stimulated BV2 Microglial Cells

**Figure 3:**
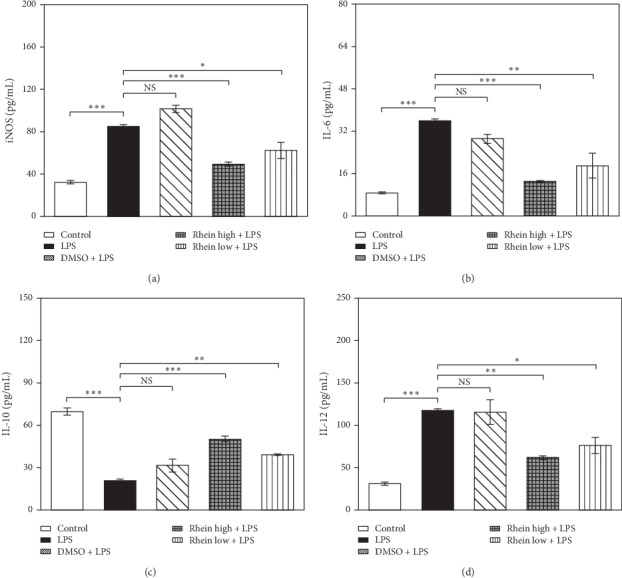
Effects of Rhein on iNOS, IL-6, IL-10, and IL-12 in LPS-stimulated BV2 cells. The cells were untreated (control) or pretreated with Rhein/DMSO for 30 min, followed by LPS stimulation (1 *μ*g/mL) for 48 h. (a-d) Effects of Rhein on supernatants iNOS, IL-6, IL-10, and IL-12 in LPS-stimulated BV2 cells, the amounts of supernatants were assayed by ELISA. Data represent the mean ± SEM of three independent experiments. ^*∗*^*p* < 0.05, ^*∗∗*^*p* < 0.01, and ^*∗∗∗*^*p* <  0.001.

We measured levels of IL-6, IL-12, iNOS, and IL-10 by ELISA tests. The results (Figure 3) exhibited an overt increase of IL-6, IL-12, and iNOS following LPS treatment. Rhein decreased these effects. Meanwhile, the production of IL-10 in LPS group was sharply cut down compared with control group. Rhein significantly heightened the level of IL-10 secretion. This drug induces an anti-inflammatory effect by inhibiting inflammatory factors and increasing the protective factor.

### 3.4. Effects of Rhein on Multiple Signaling Pathways in LPS-Caused BV2 Microglial Cells

As far as we know, the production of cytokine mediators and neurotoxic factors induced by LPS associated with multiple signaling pathways during neuroinflammation. PI3K/Akt [23], p38, and ERK1/2 [24, 25] signaling cascades are highly correlated with neuroinflammation. NF-*κ*B is the key factor for proinflammatory gene [26]. To further elucidate the signaling pathways for potential interventions of Rhein, we investigated whether Rhein inhibited neuroinflammation through multiple signaling pathways.

**Figure 4:**
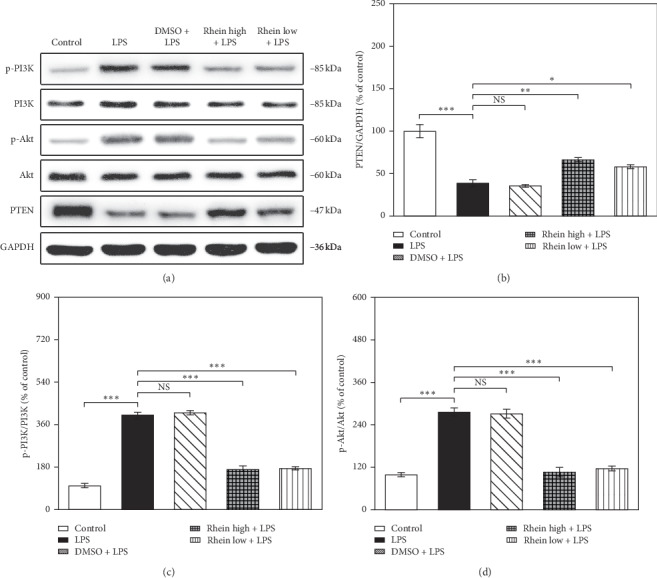
Effects of Rhein on PI3K/Akt pathway components in LPS-stimulated BV2 cells. The cells were untreated (control) or pretreated with Rhein/DMSO for 30 min, followed by LPS stimulation (1 *μ*g/mL) for 48 h. WB analysis was carried out to evaluate the protein levels of PI3K, p-PI3K, Akt, p-Akt, and PTEN. GAPDH as internal control. Data represent the mean ± SEM of three independent experiments. ^*∗*^*p* < 0.05, ^*∗∗*^*p* < 0.01, and ^*∗∗∗*^*p* <  0.001.

**Figure 5:**
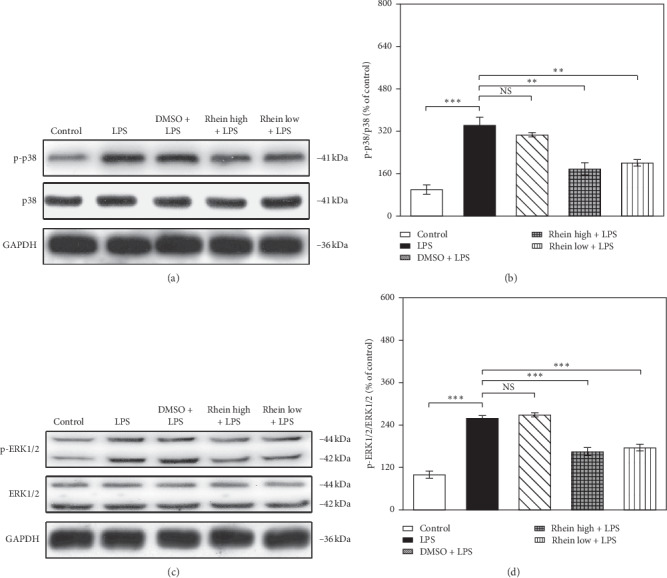
Effects of Rhein on p38 and ERK1/2 pathway components in LPS-stimulated BV2 cells. The cells were untreated (control) or pretreated with Rhein/DMSO for 30 min, followed by LPS stimulation (1 *μ*g/mL) for 48 h. WB analysis was carried out to evaluate the protein levels of p38, p-p38, ERK1/2, and p-ERK1/2. GAPDH as internal control. Data represent the mean ± SEM of three independent experiments. ^*∗∗*^*p* < 0.01 and ^*∗∗∗*^*p* <  0.001.

**Figure 6:**
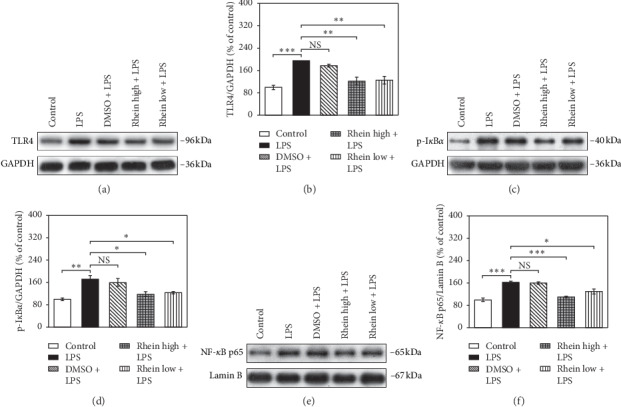
Effects of Rhein on TLR4/NF-*κ*B pathway components in LPS-stimulated BV2 cells. The cells were untreated (control) or pretreated with Rhein/DMSO for 30 min, followed by LPS stimulation (1 *μ*g/mL) for 48 h. WB analysis was carried out to evaluate the protein levels of TLR4, p-I*κ*B*α*, and NF-*κ*B p65. GAPDH as internal control for total protein and cytoplasmic protein; Lamin B as internal control for nucleoprotein. Data represent the mean ± SEM of three independent experiments. ^*∗*^*p* < 0.05, ^*∗∗*^*p* < 0.01, and ^*∗∗∗*^*p* <  0.001.

As depicted in Figures 4–6, under the stimulation with LPS, the phosphorylation levels of PI3K, Akt, p38, ERK1/2, and I*κ*B*α* were significantly upregulated (*p* < 0.01), and TLR4 and NF-*κ*B p65 protein expression was also increased. Contrarily, the protein standard of PTEN was downregulated, whereas Rhein treatment forcefully attenuated the above effects. Rhein notably led to dephosphorylation of PI3K, Akt, p38, and ERK1/2. Additionally, Rhein markedly inhibited the expression of TLR4, p-I*κ*B*α,* and NF-*κ*B p65. The augmentation in PTEN was reversed by Rhein. Thus, these observations manifested the inhibitory actions of Rhein via multiple signaling pathways (PI3K/Akt, p38, ERK1/2, and TLR4/NF-*κ*B signaling pathways) in LPS-activated BV2 cells.

## 4. Discussion

**Figure 7:**
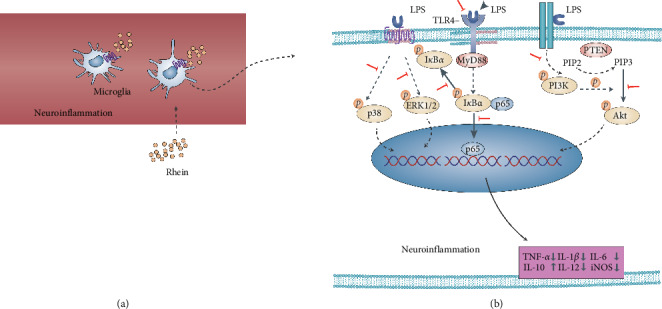
Diagrammatic sketch of Rhein as a potential therapeutic agent against neuroinflammation. Rhein significantly attenuated the proinflammatory cytokines TNF-*α*, IL-1*β*, IL-6, IL-12, and iNOS in LPS-induced BV2 cells and enhanced the anti-inflammatory cytokine IL-10. In addition, antineuroinflammatory effects are associated with inhibition of PI3K/Akt, p38, ERK1/2, and TLR4/NF-*κ*B signaling pathways.

In this work, Rhein significantly reduced the expression of inflammatory factors including TNF-*α*, IL-1*β*, IL-6, IL-12, and iNOS in in vitro model of neuroinflammation. In additional, this drug remarkably upregulated the expression of the protective factor IL-10. The potential molecular mechanisms were related to the inhibition of PI3K/Akt, p38, ERK1/2, and TLR4/NF-*κ*B signaling pathways (Figure 7).

Microglia, as the resident immunity cells, plays the role of innate immunity mediators in the CNS [20]. In the CNS pathology progress, microglia respond to pathogenic challenges and trigger their own activation and proliferation. The activated microglia cells produce immune response through secreting excess immune mediators like cytokine mediators and neurotoxic factors, causing neuroinflammation and associating neurological and neurodegenerative diseases [27]. Therefore, rapid containment of immune mediators through microglia is of utmost priority; it also becomes the central issue of the study of antineuroinflammatory ingredients to complete the drug transformation. LPS-stimulated BV2 cells were chosen as the model for screening and evaluation of antineuroinflammatory agents in this study. Since 48 h is the proliferative peak of microglia in CNS pathology progress [28], this timepoint was selected as the observation end point.

Among the immune mediators released from the activated microglia, iNOS and proinflammatory cytokines, for example, TNF-*α*, IL-1*β*, IL-6, and IL-12, are known to be major mediators in the exacerbation of neuroinflammation. In the face of various injuries and pathogenic stimuli, microglia appear to express amounts of iNOS in the CNS [29]. A wide range of evidence indicates that a series of cytokines are involved in neuroinflammatory responses, such as TNF-*α*, IL-1*β*, IL-6, IL-10, and IL-12. Among them, TNF-*α* released from microglia is the center of neuroinflammatory responses under pathological conditions [21]. As the biomarker of early neuroinflammation and brain tissue damage, IL-1*β* promotes the increase of other proinflammatory cytokines and chemokines, synergistically resulting in neurotoxicity with TNF-*α* [22]. IL-6 and IL-12 strengthen acute neuroinflammatory reactions to inflammatory injury [30, 31]. These proinflammatory cytokines become the indicators of microglia activation. In addition, as the anti-inflammatory cytokines, IL-10 limits inflammation in the brain. It is exerted by inhibiting synthesis of proinflammatory cytokines, suppressing cytokine receptor expression and activation [32]. As expected in our experiment, LPS stimulation caused a robust proinflammatory response, accompanied by the upregulation of proinflammatory cytokines and neurotoxic factors, downregulation of anti-inflammatory cytokine. We found that Rhein attenuated LPS-stimulated TNF-*α*, IL-1*β*, iNOS, IL-6, and IL-12 expression. Additionally, IL-10 content was increased after Rhein treatment. These results suggest that Rhein may possess vigorous antineuroinflammatory properties.

For the sake of probing the Rhein underlying antineuroinflammatory mechanisms, the key neuroinflammation related signaling pathways are assessed. In neuroinflammation, the signaling pathways that mediate these processes are complex and involve many coordinated kinases. Researches have shown that the activation of the PI3K/Akt, p38, ERK1/2, and TLR4/NF-*κ*B signaling pathways is primarily involved following neuroinflammation [23–26].

PI3K/Akt signaling pathway has been reported to have a role in regulating acute inflammation [33]. Once activated, PI3K phosphorylates phosphatidylinositol-4,5-diphosphate (PIP2) to form phosphatidylinositol-3,4,5-triphosphate (PIP3). Then PIP3 catalyses Akt, and phosphorylated Akt activates downstream proteases, which in turn secrete inflammatory factors and mediate inflammatory responses [34]. PTEN is a pivotal regulator of PI3K/Akt signaling pathway; it dephosphorylates PIP3 on the membrane to PIP2 [35]. We found that Rhein reduced phosphorylated PI3K and Akt, increasing PTEN expression when compared to LPS-treated group. The findings suggest that Rhein tends to inhibit PI3K/Akt signaling pathway against neuroinflammation.

Similarly, p38 and ERK1/2 play crucial roles in signaling events that contribute to the production of neuroinflammatory mediators. As the core of neuroinflammatory response, p38 was rapidly phosphorylated in response to LPS. ERK1/2 is a potent effector of neuroinflammation in CNS diseases [24]. The p38-activated transduction regulates the levels of TNF-*α*, IL-1*β*, IL-6, and IL-10 [36]. Signal transduction by ERK1/2 activation contributes to the release of cytokines such as IL-1*β*. In our experiments, p38 and ERK1/2 were phosphorylated by LPS stimulation, while Rhein pretreatment restrained the phosphorylation of p38 and ERK1/2. Thus, the regulation of Rhein on the production of neuroinflammatory mediators seems to be achieved by suppressing p38 and ERK1/2 activations.

TLR4/NF-*κ*B signaling pathway acts major player of neuroinflammatory response following LPS stimulation. TLR4 specifically recognizes LPS, causing I*κ*B*α* phosphorylation and degradation. NF-*κ*B which bonds to I*κ*B*α* dissociates from the complex and shifts to the nucleus, resulting in its own activation [37]. Activated NF-*κ*B combines with the promoter regions of inflammatory molecules to increase inflammation-associated genes expression, ultimately contacting the release of inflammatory mediators, including TNF-a, IL-1, IL-6, and iNOS [38]. Our work reveals that LPS stimulation leads to the activation of TLR4 and I*κ*B*α*, as well as nuclear transfer of NF-*κ*B. Rhein can inhibit the abovementioned conditions in LPS-mediated BV2 cells. Therefore, in LPS-stimulated BV2 cells, the antineuroinflammatory effects of Rhein are partially composed of the TLR4/NF-*κ*B signaling pathway inactivation.

## 5. Conclusions

In summary, the present study displays the anti-inflammatory effects of Rhein in LPS-induced BV2 cells. Our research suggests that Rhein tends to be a potential therapeutic agent for the prevention and treatment of neuroinflammation. This work highlights Rhein as an effective neuroprotective agent for antineuroinflammation.

## Data Availability

The data used to support the findings of this study are available from the corresponding author upon request.
